# Supplementary material for: A Gamma-adapted subunit vaccine induces broadly neutralizing antibodies against SARS-CoV-2 variants and protects mice from infection
Source: Nat Commun. 2024 Feb 2;15:997. doi: 10.1038/s41467-024-45180-8 (PMC10837449; doi:10.1038/s41467-024-45180-8)
Supplement: Supplementary file 1 — Supplementary Information [file 41467_2024_45180_MOESM1_ESM.pdf]

Title: A Gamma-adapted subunit vaccine induces broadly neutralizing antibodies against SARS-CoV-2 variants and protects mice from infection.

SUPPLEMENTARY INFORMATION

Supplementary Figure 1.

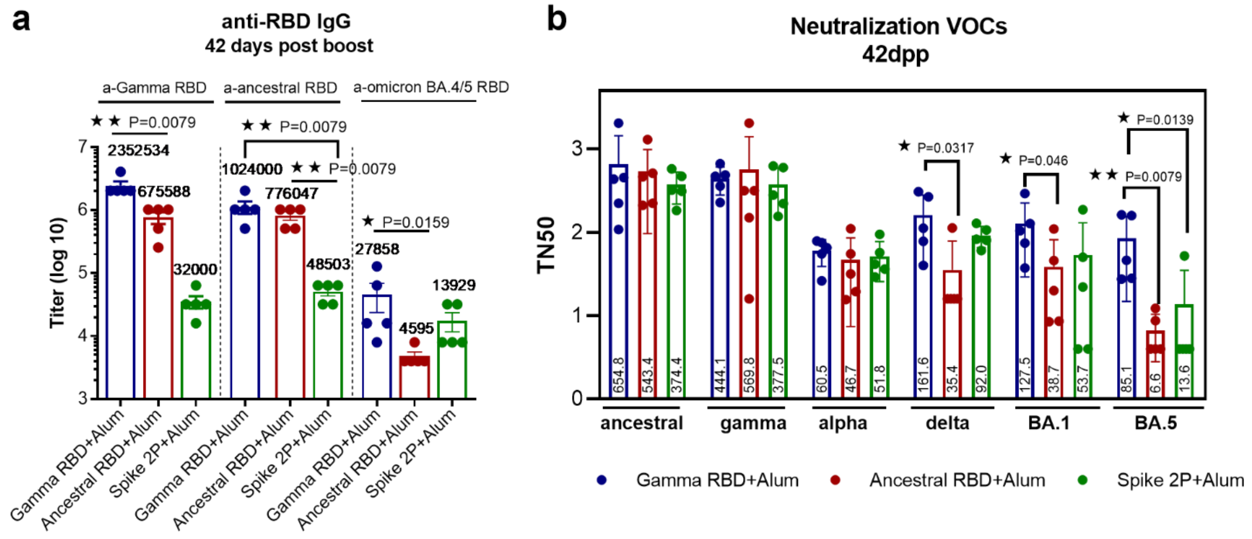

Supplementary Figure 1. Antibody responses induced by different recombinant vaccine formulations in mice. BALB/c mice were immunized i.m with i) Gamma RBD+Alum (blue circles, n= 5 mice), ii) Ancestral RBD+Alum (red circles, n= 5 mice) or iii) Spike 2P+Alum (green circles, n= 5 mice) on days 0 and 14. **a**. At day 42 specific antibody responses against RBD (Gamma, Ancestral or Omicron BA.4/5) were evaluated in serum by ELISA. Error Bars are Mean ± SD values. Kruskal Wallis test. *p<0.05, **p<0.01. Exact P values are shown. **b**. Neutralizing antibody response in serum were measured at 42 days post prime immunization (dpp). Neutralization titers against SARS-CoV-2 variants (Ancestral, Gamma, Alpha, Delta, Omicron BA.1 and BA.5) was determined by live virus assay. Neutralization titer was defined as the serum dilution that reduces 50% of the cytopathic effect (NT50). Points represent data of individual mice. Error Bars are GMT ± SD values. Kruskal Wallis test. *p<0.05, **p<0.01. Exact P values are shown. Representative of two independent experiments. Source data are provided as a Source Data file.

Supplementary Figure 2.

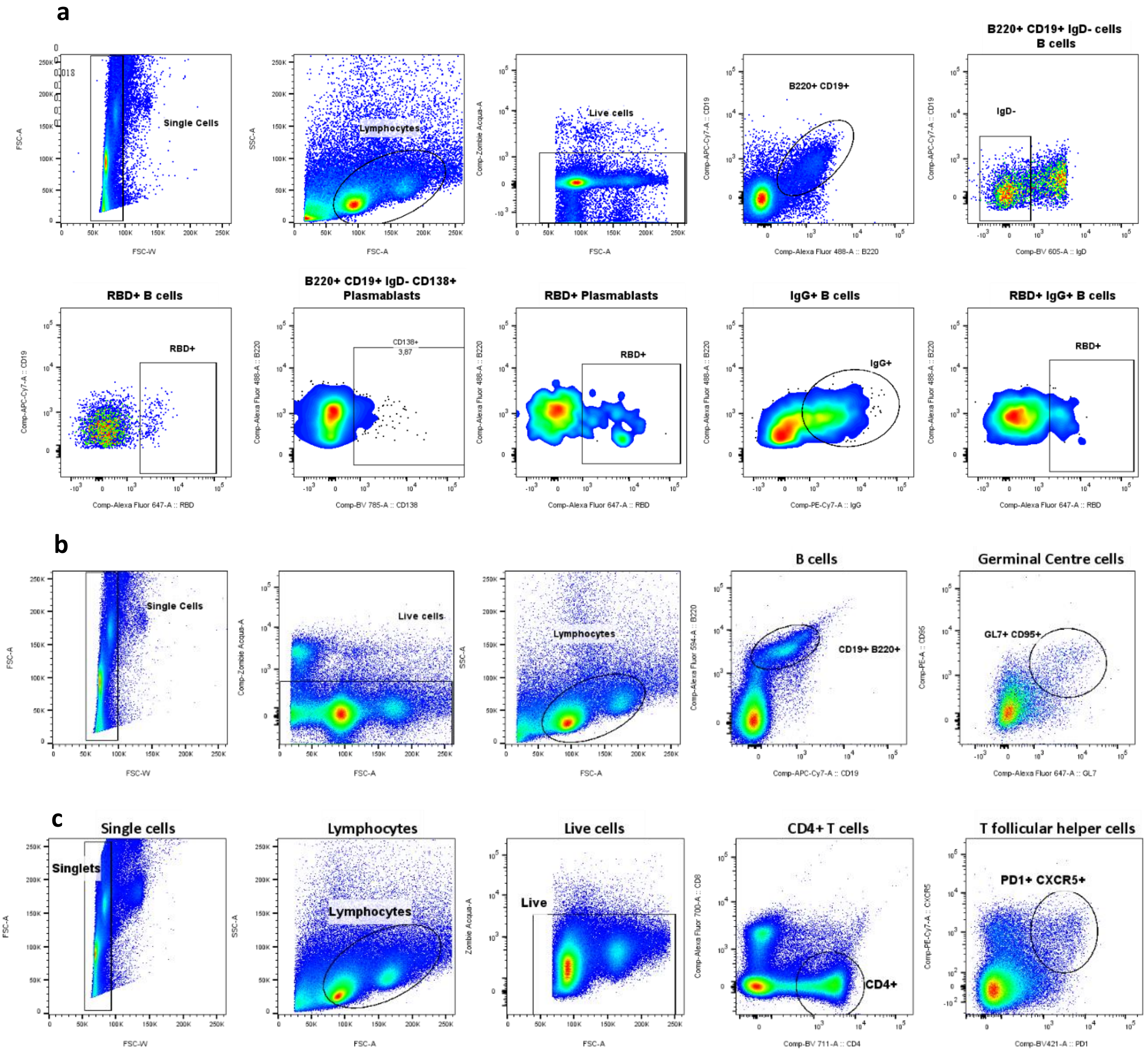

Supplementary Figure 2. Gating strategy of flow cytometry results presented in Figure 2. **a**. Gating strategy for the analysis of B cells and plasmablasts in spleen presented inf Fig.2e. Dot plots with gates are shown in sequential order. **b**. Gating strategy for the analysis of Germinal Centre cells in spleen presented in figure 2f. Dot plots with gates are shown in sequential order. **c**. Gating strategy for analysis of T follicular helper cells in spleen presented in figure 2g. Dot plots with gates are shown in sequential order.

Supplementary Figure 3.

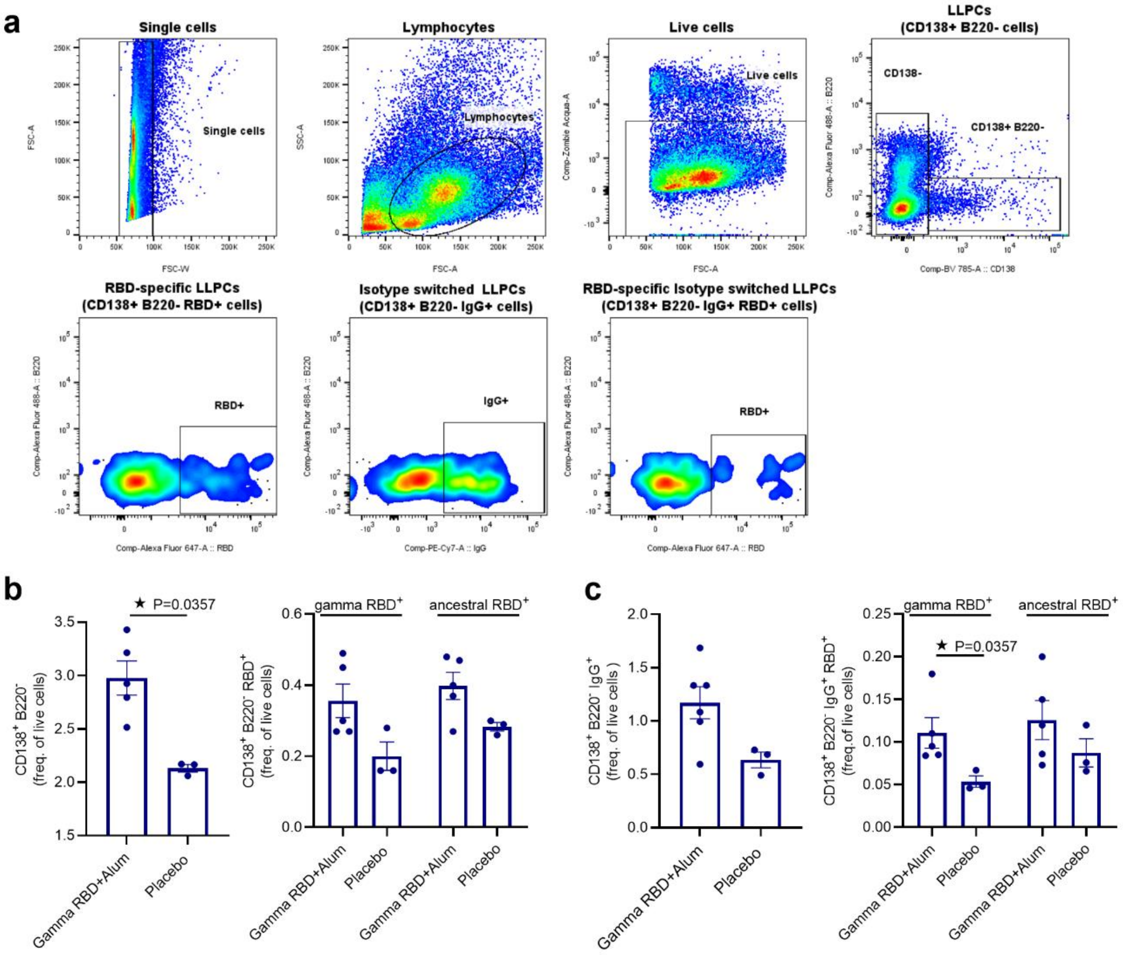

Supplementary Figure 3. Long-lived plasma cells are present in bone marrow after mice immunization with the Gamma RBD vaccine. BALB/c mice were immunized i.m with Gamma RBD+Alum (n=5 mice) or placebo (n= 3 mice) on days 0 and 14.**a**. Gating strategy for evaluation of LLPCs and isotype-switched LLPCs specific for RBD. **b**. LLPCs were evaluated in the bone marrow at day 42 after mice immunization by flow cytometry. Cells suspensions were obtained, and live cells were stained with Zombie Acqua dye, then specific mAbs against B220, CD138 and IgG were used. Specificity was evaluated labelling cells with a fluorescent RBD antigen. Total ($CD138^+$ $B220^-$) or specific ($CD138^+$ $B220^-$ RBD^+) LLPCs and total ($CD138^+$ $B220^-$ IgG^+) or specific ($CD138^+$ $B220^-$ IgG^+ RBD^+) isotype-switched LLPCs frequencies are shown. Points are frequencies of the live cell population for each mouse. Error bars are means ± SEM values. Two-sided Mann Whitney test. *p<0.05 Exact P value is shown. Source data are provided as a Source Data file.

Supplementary Figure 4.

a. Gating strategy used for the evaluation of cytokine producing T cells:

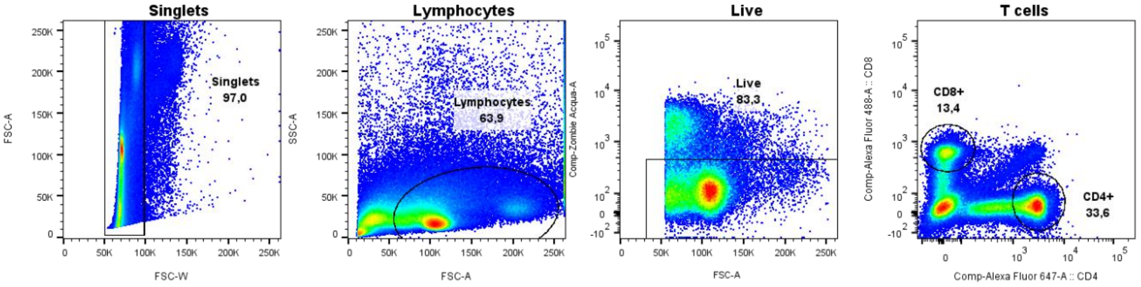

b. Representative dot plots of the FMO control.

FMO control (only include Zombie Acqua dye and anti-CD4 and CD8 antibodies.)

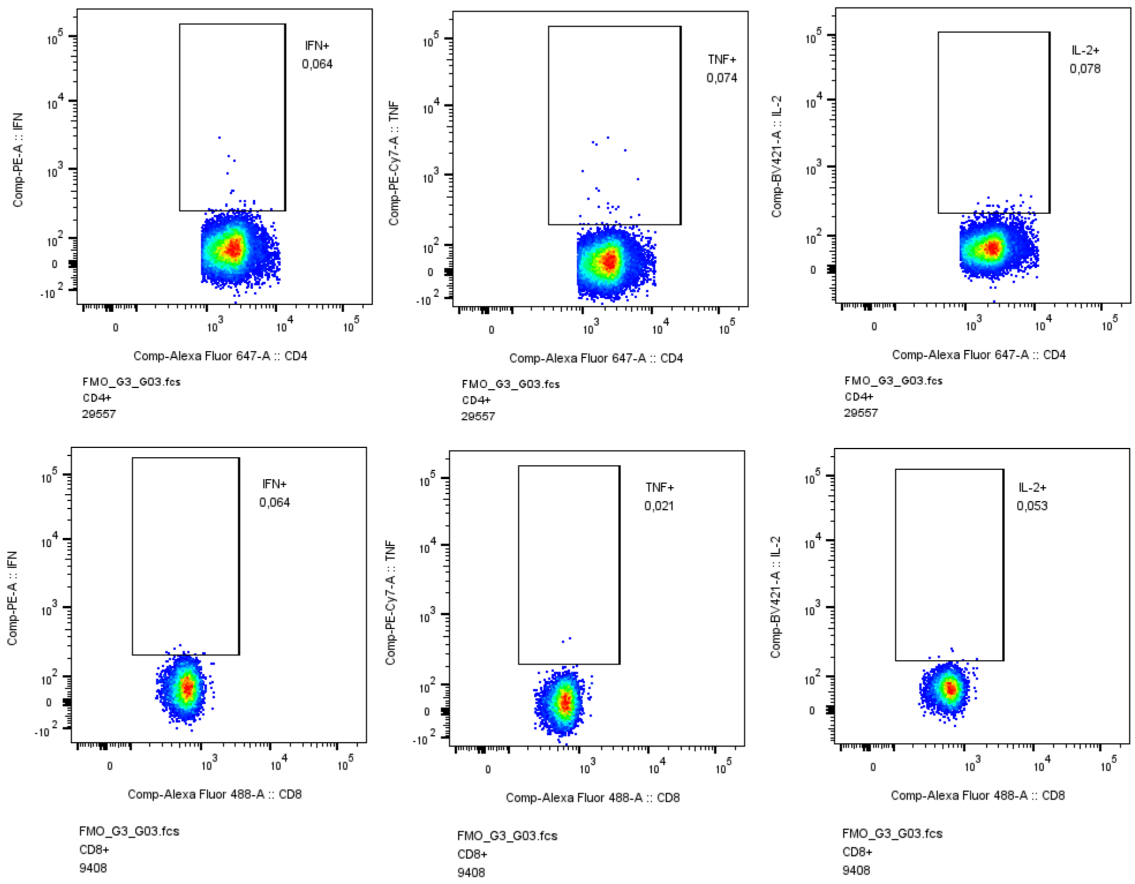

c. **Representative dot plots of the flow cytometry data shown in Figure 4a.**

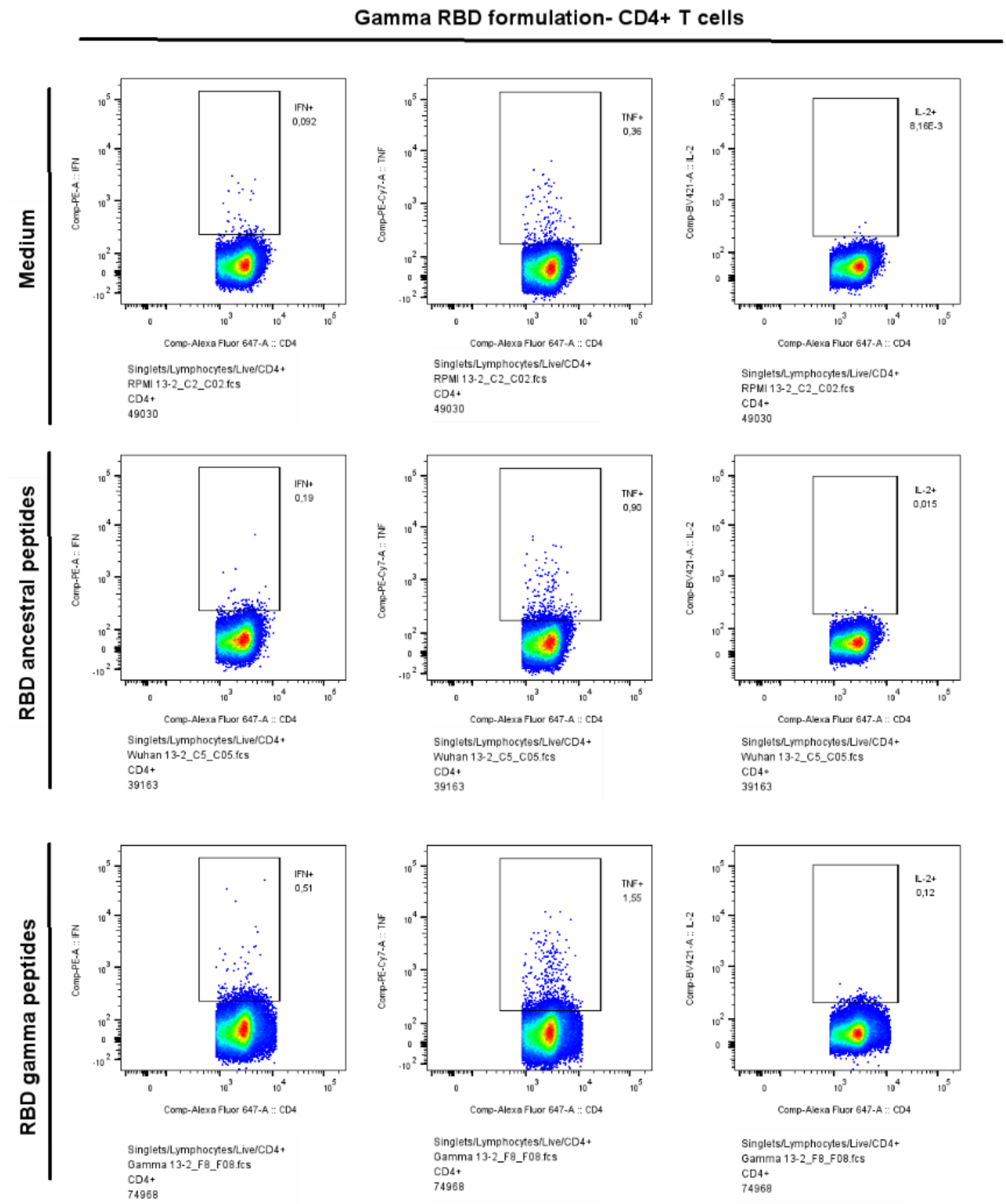

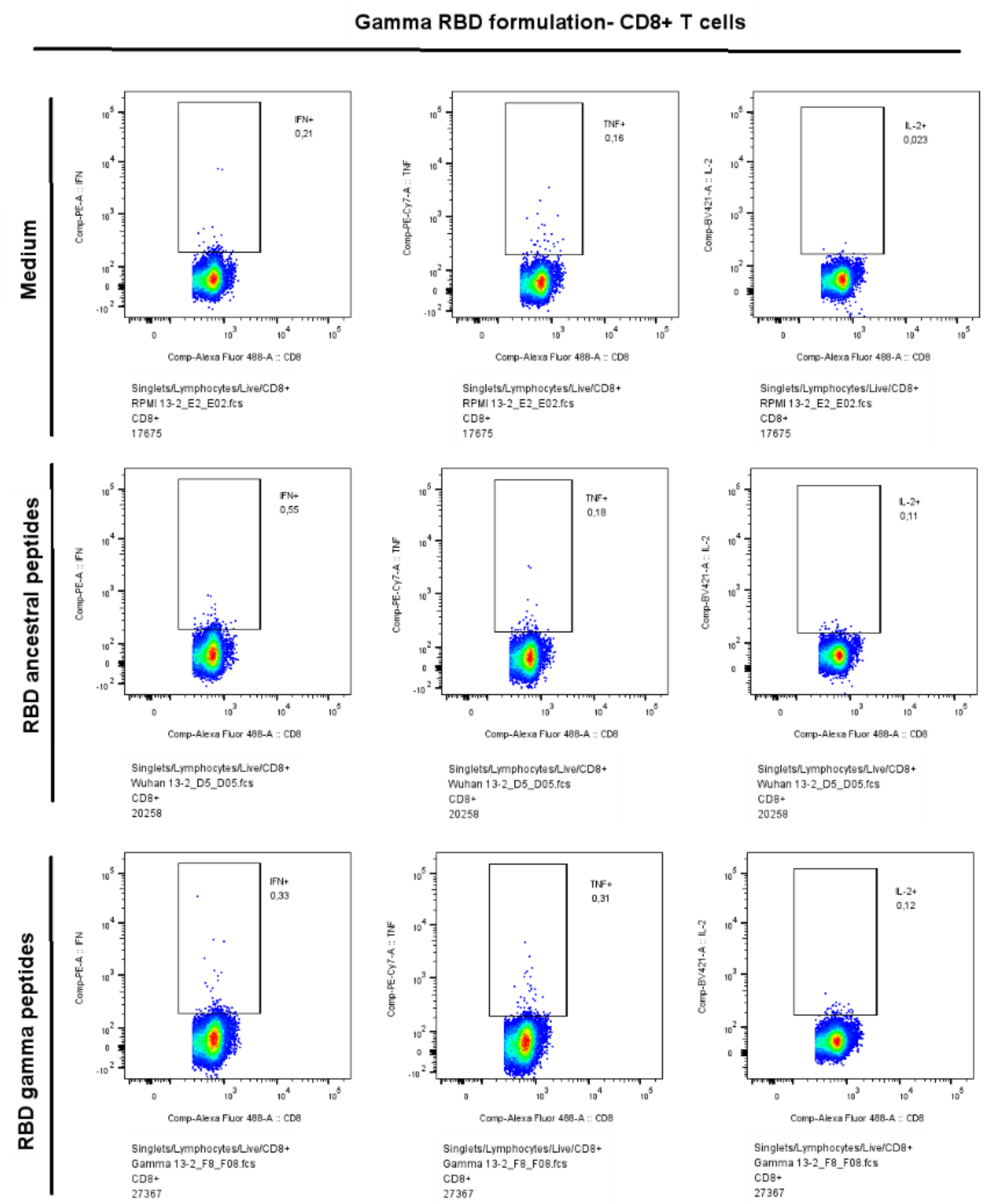

Gamma RBD formulation- CD8+ T cells
Medium
RBD ancestral peptides
RBD gamma peptides
Comp-PE-A :: IFN
Comp-PE-Cy7-A :: TNF
Comp-BV421-A :: IL-2
Comp-Alexa Fluor 488-A :: CD8
IFN+ 0,21
TNF+ 0,16
IL-2+ 0,023
Singlets/Lymphocytes/Live/CD8+
RPMI 13-2_E2_E02.fcs
CD8+
17675
IFN+ 0,55
TNF+ 0,18
IL-2+ 0,11
Singlets/Lymphocytes/Live/CD8+
Wuhan 13-2_D5_D05.fcs
CD8+
20258
IFN+ 0,33
TNF+ 0,31
IL-2+ 0,12
Singlets/Lymphocytes/Live/CD8+
Gamma 13-2_F8_F08.fcs
CD8+
27367

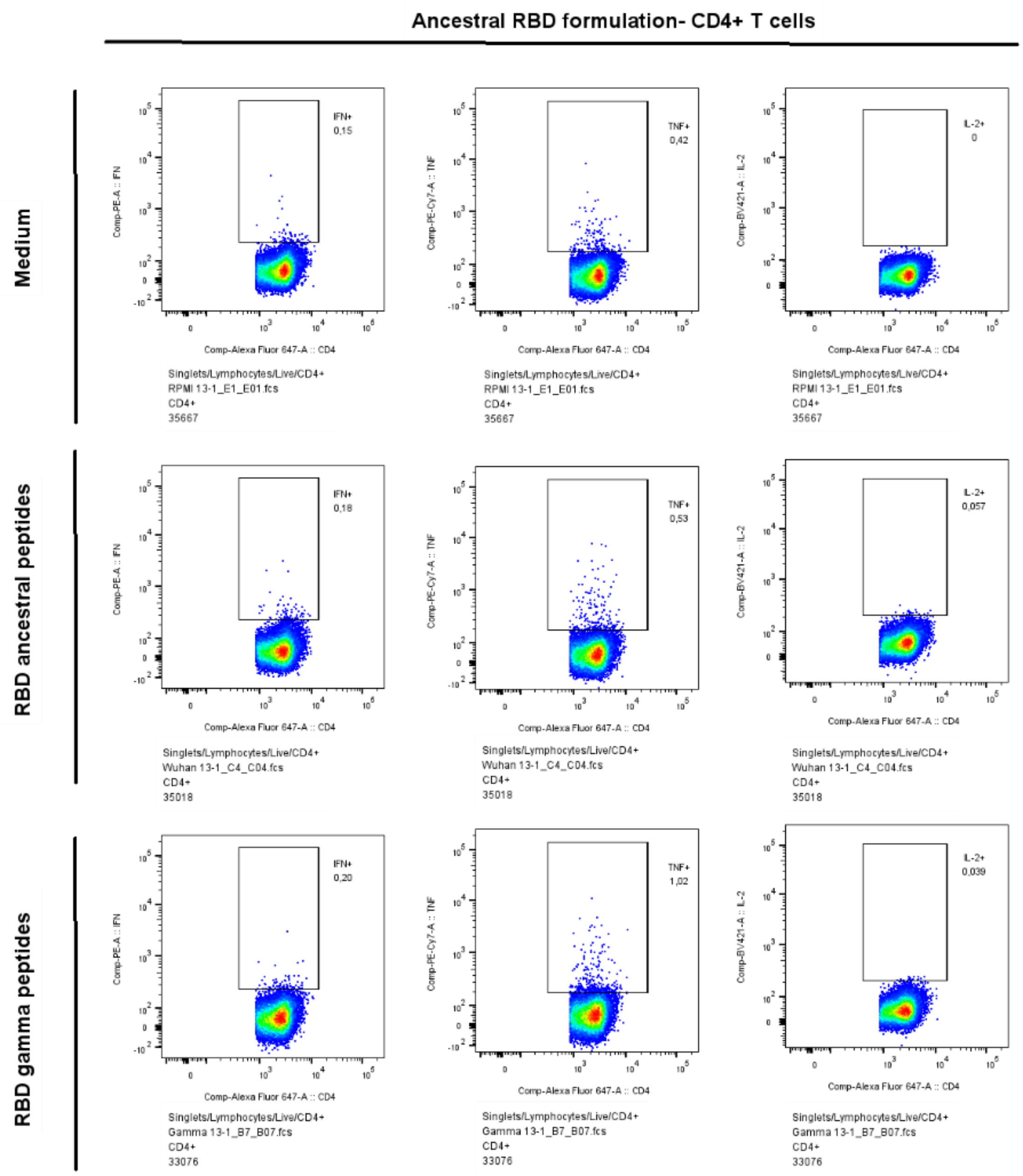
Ancestral RBD formulation- CD4+ T cells
Medium
RBD ancestral peptides
RBD gamma peptides
IFN+
0,15
TNF+
0,42
IL-2+
0
IFN+
0,18
TNF+
0,53
IL-2+
0,057
IFN+
0,20
TNF+
1,02
IL-2+
0,039
Comp-PE-A :: IFN
Comp-PE-Cy7-A :: TNF
Comp-BV421-A :: IL-2
Comp-Alexa Fluor 647-A :: CD4
Singlets/Lymphocytes/Live/CD4+
RPMI 13-1_E1_E01.fcs
CD4+
35667
Singlets/Lymphocytes/Live/CD4+
Wuhan 13-1_C4_C04.fcs
CD4+
35018
Singlets/Lymphocytes/Live/CD4+
Gamma 13-1_B7_B07.fcs
CD4+
33076

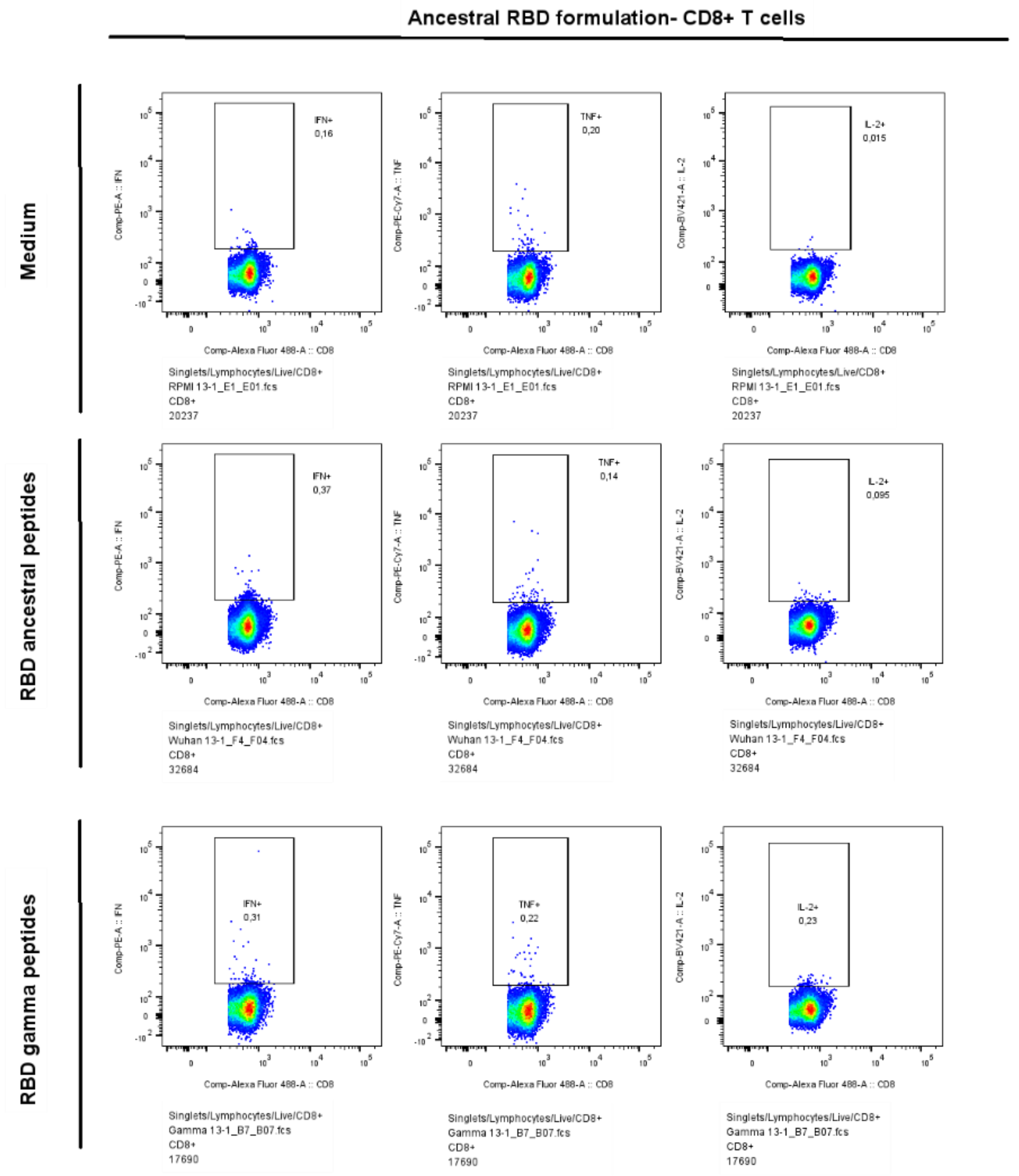

Supplementary Figure 4. Gating strategy of intracellular flow cytometry results presented in Figure 4a. **a**. Gating strategy used for the evaluation of cytokine producing CD4$^+$ and CD8$^+$ T cells. Dot plots with gates are shown in sequential order**. b**. Dot plots of the fluorescence minus one (FMO) control used to set negative populations of cytokine producing CD4$^+$ and CD8$^+$ T cells. **c**. Representative dot plots of the intracellular flow cytometry data shown in Figure 4a. Representative dot plots for each vaccinated group and each stimulus condition (medium, RBD ancestral peptides and RBD gamma peptides) is shown for each cytokine (IFN-γ, TNF-α and IL-2).

Supplementary Figure 5

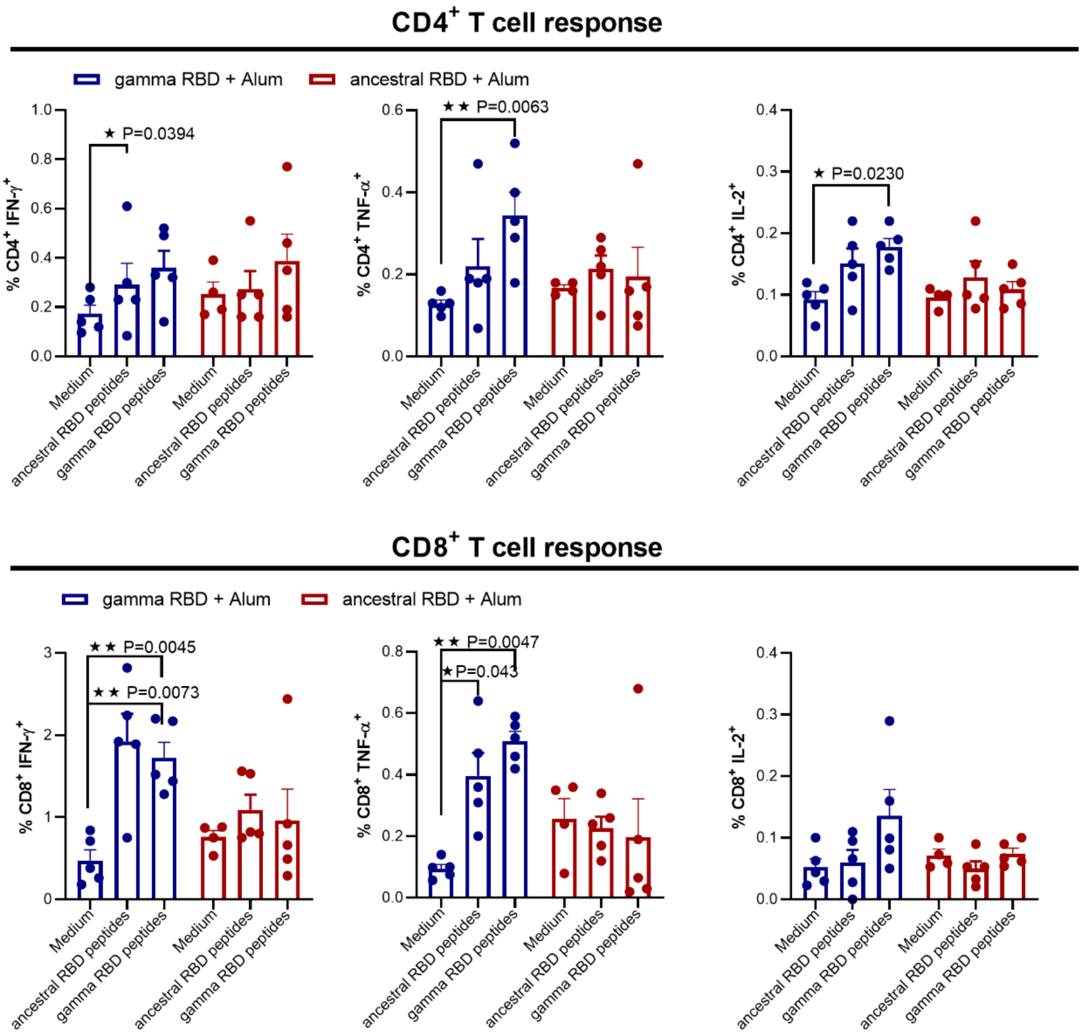

Supplementary Figure 5. T cell immune responses elicited in C57BL/6 mice after vaccination with the Gamma and Ancestral RBD adjuvanted formulations. C57BL/6 mice were i.m. inoculated on days 0 and 14 with Gamma RBD + Alum (blue circles, n=5 mice) or Ancestral RBD + Alum (red circles, n=5 mice). Cellular responses were evaluated in spleen 28 days after last immunization. **Intracellular flow cytometry analysis of cytokine secreting T cells**. Splenocytes were stimulated with complete media or a peptide pool derived from RBD (gamma or ancestral) and then brefeldin A was added. Afterward, cells were harvested and stained with specific Abs including anti-CD8, and anti-CD4, fixed, permeabilized, and stained intracellularly with anti–IFN-γ, TNF-α and anti-IL-2. Results are presented as percentage of cytokine-producing $CD4^{+}$ (upper panel) or $CD8^{+}$ (lower panel) T cells. Bars are means ± SEM. *$p < 0.05$ vs. medium. **$p < 0.01$ vs. medium. Exact p values are shown. Kruskal Wallis test. Source data are provided as a Source Data file.

Supplementary Figure 6.

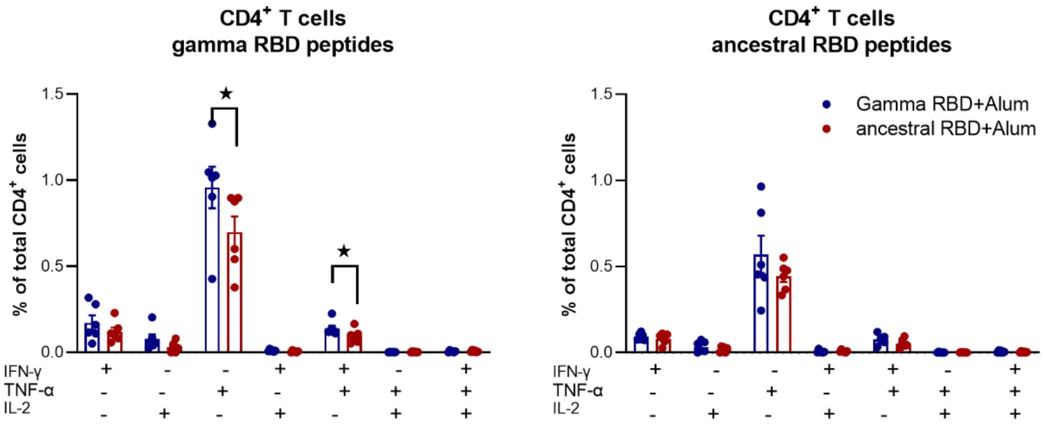

Supplementary Figure 6. Assessment of polyfunctional T cells induced by vaccine formulations. BALB/c mice were i.m. inoculated on days 0 and 14 with Gamma RBD + Alum (blue circles, n=5 mice) + Ancestral RBD + Alum (red circles, n=5 mice). Frequency of $CD4^+$ T cell populations from splenocytes that produce one, two or three cytokines after gamma (left panel) or ancestral (right panel) RBD peptides stimulation were plotted for each group of mice Bars are means ± SEM. *P=0.0411. Kruskal Wallis test. Representative of two independent experiments. Source data are provided as a Source Data file.

Supplementary Figure 7.

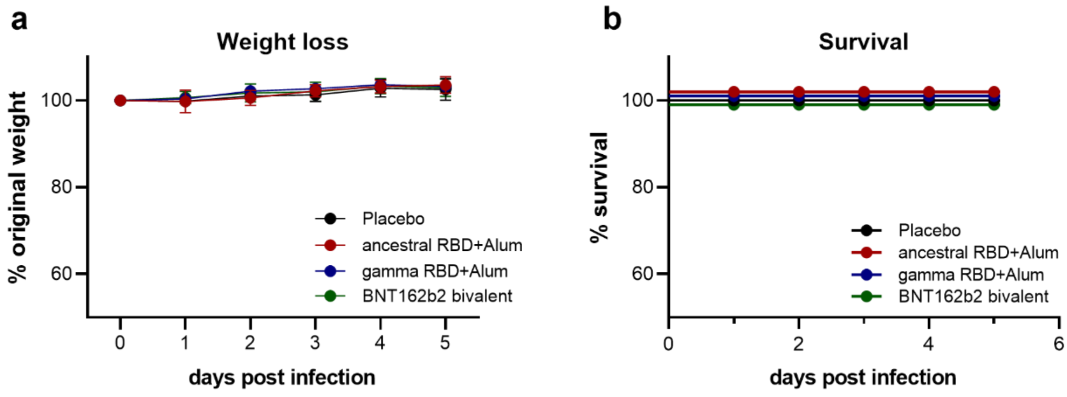

Supplementary Figure 7. K18-hACE2 transgenic mice weight loss and survival after intranasal BA.5 SARS-CoV-2 infection. Mice were i.m. inoculated on days 0 and 14 with: i) placebo (n=12 mice, black circles), ii) Gamma RBD + Alum (n=12 mice, blue circles), iii) Ancestral RBD + Alum (n=12 mice, red circles) or iv) bivalent BNT162b2 (n=12, green circles). Two weeks following immunization, mice were intranasally infected with 1× 10^4 PFU of Omicron BA.5. **a**. Weight changes in mice were monitored daily until day 5 after infection. Points are means ± SEM of percentage of original weight. **b**. Survival were monitored daily until day 5. Each point represents the percentage of mice alive at that time. Source data are provided as a Source Data file.

Supplementary Figure 8.

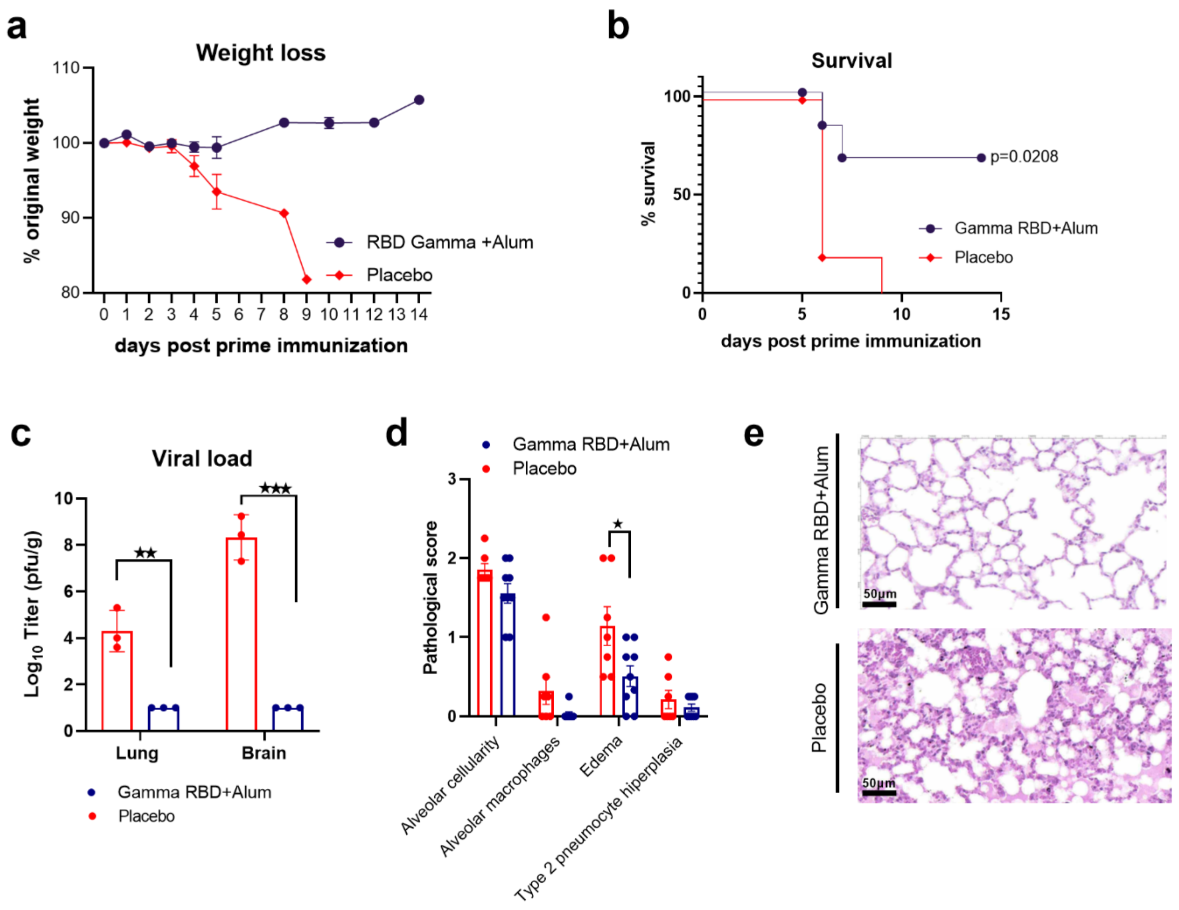

Supplementary Figure 8. Vaccination with Gamma RBD + Alum protects K18-hACE2 transgenic mice against intranasal SARS-CoV-2 infection. Mice received PBS (Control, n=8 mice) or Gamma RBD + Alum (n=9 mice) administered via i.m. route on days 0 and 14. Four weeks following immunization, K18-hACE2 mice were intranasally infected with 2×10^5 PFU of SARS-CoV-2. **a**. Weight changes in mice were monitored daily until day 14 after infection. Points are means ± SEM of percentage of original weight. **b**. Survival were monitored daily until day 14. Each point represents the percentage of mice alive at that time. Survival curves were analyzed with Log-rank (Mantel-Cox) test. P=0.0208 vs placebo. **c**. Five days after infection lungs and brains (n=3) were obtained from groups of mice and SARS-CoV-2 virus was titrated. Bars represent the mean ± SEM. **P < 0.01 and ***P < 0.001. Unpaired T test. **d.** Histopathologic studies were performed on lung samples from the indicated groups, and pathological scores were calculated for each parameter (alveolar cellularity, alveolar macrophages, edema and type 2 pneumocyte hyperplasia). Statistical analysis was conducted using Two-sided Mann-Whitney test. *P < 0.05. **e**. Hematoxylin and eosin staining of lung sections from K18-hACE2 infected mice at 5 dpi. Scale bars=50 µm. Source data are provided as a Source Data file.

Supplementary Table 1. Antigenic distances calculated from vaccinated mouse sera obtained 28 days after the second dose. 1 unit of antigenic distance is equivalent to a two-fold dilution in neutralization titers. The lowest distance among the groups is colored in grey.

Antigenic distances		
SARS-CoV-2 variants	Gamma RBD vaccine	Ancestral RBD vaccine
Ancestral-Gamma	0.60	1.87
Ancestral-Alpha	3.56	3.69
Ancestral-Delta	2.35	4.68
Ancestral-Omicron BA.1	2.62	4,50
Ancestral-Omicron BA.5	3.45	6,28
Gamma-Alpha	2.96	5.19
Gamma-Delta	1.94	4.49
Gamma-Omicron BA.1	2.33	3.79
Gamma-Omicron BA.5	2.86	8.14
Alpha-Delta	2.05	8.16
Alpha-Omicron BA.1	3.42	8.16
Alpha-Omicron BA.5	0.86	7.10
Delta-Omicron BA.1	3.76	1.32
Delta-Omicron BA.5	1.42	6.12
Omicron BA.1-Omicron BA.5	3.89	7.22

Supplementary Table 2. Best linear (continuous) B-cell epitopes of the RBD of SARS-CoV-2 spike protein (Ancestral and Gamma variant) predicted by IEDB analysis of resources. BepiPred 2.0 was used to predict the linear B-cell epitopes of the RBD domain with a threshold of 0.5. Sequence of linear B cell epitopes, starting and end position, score and antigenicity predicted by Vaxijen 2.0 is shown. Residue substitution N501Y in the Gamma VOC sequence is shown in red. NA: no antigenic. A: antigenic. RBD protein sequence correspond to residues 319 to 537 of SARS-CoV-2 spike protein.

No.	Start position	End position	Peptide	Length	Score	Antigenicity
**-Ancestral RBD sequence**						
3	344	349	ATRFAS	6	0.1511	NA
4	351	363	YAWNRKRISNCVA	13	0.3936	NA
5	372	378	ASFSTFK	7	0.0865	NA
9	494	506	SYGFQPTNGVGYQ	13	0.7632	A
10	524	534	VCGPKKSTNLV	11	0.1358	NA
**-Gamma RBD sequence**						
3	344	349	ATRFAS	6	0.1511	NA
4	351	363	YAWNRKRISNCVA	13	0.3936	NA
5	372	378	ASFSTFK	7	0.0865	NA
9	496	506	SYGFQPT**Y**GVGYQ	13	**0.8457**	A

10	524	534	VCGPKKSTNLV	11	0.1358	NA

Supplementary Table 3. In silico screening of MHC class II restricted T-cell based epitopes of the RBD protein from SARS-CoV-2. Using netMHCpan 3.2 MHC-II (I-A/E) restricted epitopes of 9 residues in length in the two more common mouse haplotypes (d and b) were selected and scored by Vaxijen 2.0. WB= Weak binding. SB= Strong Binding. New epitopes generated are colored in grey.

MHCII allelles

I-Ad

		ancestral RBD antigen			gamma RBD antigen		
**Position**	**Peptide**	**Score**	**Binding**	**VaxiJen**	**Score**	**Binding**	**VaxiJen**
20	FGEVFNATRFASVYA	6.00	WB	0.0415 (NA)	6.00	WB	0.0415 (NA)
21	GEVFNATRFASVYAW	4.00	WB	-0.1202 (NA)	4.00	WB	-0.1202 (NA)
22	EVFNATRFASVYAWN	4.00	WB	0.0832 (NA)	4.00	WB	0.0832 (NA)
23	VFNATRFASVYAWNR	3.00	WB	0.0745 (NA)	3.00	WB	0.0745 (NA)
24	FNATRFASVYAWNRK	3.50	WB	0.4491 (A)	3.50	WB	0.4491 (A)
25	NATRFASVYAWNRKR	5.50	WB	0.4062 (A)	5.50	WB	0.4062 (A)
26	ATRFASVYAWNRKRI	7.00	WB	0.3489 (NA)	7.00	WB	0.3489 (NA)
46	DYSVLYNSASFSTFK	9.50	WB	0.2080 (NA)	9.50	WB	0.2080 (NA)
47	YSVLYNSASFSTFKC	9.50	WB	0.1176 (NA)	9.50	WB	0.1176(NA)
192	VVVLSFELLHAPATV	9.00	WB	0.8083 (A)	9.00	WB	0.8083 (A)
193	VVLSFELLHAPATVC	8.00	WB	0.8618 (A)	8.00	WB	0.8618 (A)
194	VLSFELLHAPATVCG	8.00	WB	0.4784 (A)	8.00	WB	0.4784(A)
195	LSFELLHAPATVCGP	9.00	WB	0.5062 (A)	9.00	WB	0.5062 (A)

I-Ed

		ancestral RBD antigen		gamma RBD antigen			
**Position**	**Peptide**	**Score**	**Binding**	**VaxiJen**	**Score**	**Binding**	**VaxiJen**
19	PFGEVFNATRFASVY	9.00	WB	0.0331 (NA)	9.00	WB	0.0331 (NA)
20	FGEVFNATRFASVYA	7.50	WB	0.0415(NA)	7.50	WB	0.0415(NA)
21	GEVFNATRFASVYAW	9.00	WB	-0.1202 (NA)	9.00	WB	-0.1202 (NA)
23	VFNATRFASVYAWNR	8.00	WB	0.0745 (NA)	8.00	WB	0.0745 (NA)
24	FNATRFASVYAWNRK	7.50	WB	0.4491 (A)	7.50	WB	0.4491 (A)
25	NATRFASVYAWNRKR	4.00	WB	0.4062 (A)	4.00	WB	0.4062 (A)
26	ATRFASVYAWNRKRI	1.20	SB	0.3489 (NA)	1.20	SB	0.3489 (NA)
27	TRFASVYAWNRKRI	0.60	SB	0.3964 (NA)	0.60	SB	0.3964 (NA)
28	RFASVYAWNRKRISN	0.50	SB	0.4243 (A)	0.50	SB	0.4243 (A)
29	FASVYAWNRKRISNC	0.90	SB	0.3676 (NA)	0.90	SB	0.3676 (NA)
30	ASVYAWNRKRISNCV	1.10	SB	0.3086 (NA)	1.10	SB	0.3086 (NA)
31	SVYAWNRKRISNCVA	1.60	SB	0.3301 (NA)	1.60	SB	0.3301 (NA)
32	VYAWNRKRISNCVAD	4.00	WB	0.1768 (NA)	4.00	WB	0.1768 (NA)
125	KVGGNYNYLYRLFRK	3.50	WB	-0.2292 (NA)	3.50	WB	-0.2292 (NA)
126	VGGNYNYLYRLFRKS	1.30	SB	-0.0193 (NA)	1.30	SB	-0.0193 (NA)
127	GGNYNYLYRLFRKSN	0.80	SB	0.0207 (NA)	0.80	SB	0.0207 (NA)
128	GNYNYLYRLFRKSNL	0.30	SB	0.1801 (NA)	0.30	SB	0.1801 (NA)
129	NYNYLYRLFRKSNLK	0.15	SB	0.1089 (NA)	0.15	SB	0.1089 (NA)
130	YNYLYRLFRKSNLKP	0.15	SB	0.2254 (NA)	0.15	SB	0.2254 (NA)
131	NYLYRLFRKSNLKPF	0.12	SB	0.0415 (NA)	0.12	SB	0.0415 (NA)
132	YLYRLFRKSNLKPFE	0.20	SB	0.0814 (NA)	0.20	SB	0.0814 (NA)
133	LYRLFRKSNLKPFER	0.40	SB	-0.0294 (NA)	0.40	SB	-0.0294 (NA)
134	YRLFRKSNLKPFERD	1.00	SB	0.1994 (NA)	1.00	SB	0.1994 (NA)
135	RLFRKSNLKPFERDI	3.50	WB	0.1391 (NA)	3.50	WB	0.1391 (NA)
136	LFRKSNLKPFERDIS	8.50	WB	0.4350 (A)	8.50	WB	0.4350 (A)

I-Ab

		ancestral RBD antigen			gamma RBD antigen		
**Position**	**Peptide**	**Score**	**Binding**	**VaxiJen**	**Score**	**Binding**	**VaxiJen**
18	CPFGEVFNATRFASV	5.00	WB	0.2975	3.00	WB	0.2975
19	PFGEVFNATRFASVY	4.00	WB	0.0331	1.70	SB	0.0331
20	FGEVFNATRFASVYA	3.50	WB	0.0415	1.20	SB	0.0415
21	GEVFNATRFASVYAW	3.50	WB	-0.1202	1.20	SB	-0.1202
22	EVFNATRFASVYAWN	5.00	WB	0.0832	1.30	SB	0.0832
23	VFNATRFASVYAWNR				1.60	SB	0.0745
24	FNATRFASVYAWNRK				3.00	WB	0.4491
25	NATRFASVYAWNRKR				6.00	WB	0.4062
26	NATRFASVYAWNRKR				7.00	WB	0.4062
27	TRFASVYAWNRKRIS	7.00	WB	0.4963	10.00	WB	0.4963
28	RFASVYAWNRKRISN	8.50	WB	0.4243			
29	FASVYAWNRKRISNC	9.50	WB	0.3676			
30	ASVYAWNRKRISNCV	9.50	WB	0.3086			
44	VADYSVLYNSASFST	8.00	WB	0.2729	5.50	WB	0.2729
45	ADYSVLYNSASFSTF	5.00	WB	0.2252	3.00	WB	0.2252
46	DYSVLYNSASFSTFK	4.50	WB	0.2080	2.50	WB	0.2080
47	YSVLYNSASFSTFKC	5.00	WB	0.1176	3.00	WB	0.1176
48	SVLYNSASFSTFKCY	6.50	WB	0.1871	4.50	WB	0.1871
49	VLYNSASFSTFKCYG	9.00	WB	0.1776	7.50	WB	0.1776
55	SFSTFKCYGVSPTKL	8.00	WB	0.9327	6.00	WB	0.9327
56	FSTFKCYGVSPTKLN	4.00	WB	1.0042	5.00	WB	1.0042
57	STFKCYGVSPTKLND	4.50	WB	1.2022	6.00	WB	1,2022
58	TFKCYGVSPTKLNDL	5.50	WB	1.4626	6.00	WB	1.4626
59	FKCYGVSPTKLNDLC	8.00	WB	1,7128			
149	DISTEIYQAGSTPCN	3.50	WB	0.1568	5.00	WB	0.1568
150	ISTEIYQAGSTPCNG	3.00	WB	-0.1516	4.00	WB	-0.1516
151	STEIYQAGSTPCNGV	3.00	WB	-0.0513	2.50	WB	-0.0513
152	TEIYQAGSTPCNGVE	3.00	WB	-0.0028	3.00	WB	-0.0544
153	EIYQAGSTPCNGVEG	4.00	WB	-0.0305	4.00	WB	0.0218
154	IYQAGSTPCNGVEGF	5.50	WB	-0.1715	6.50	WB	-0.1477
171	YFPLQSYGFQPTNGV	9.50	WB	0.6881			
172	FPLQSYGFQPTNGVG	9.00	WB	0.5697			
173	PLQSYGFQPTNGVGY	7.50	WB	0.3415	6.50	WB	0.4135
174	LQSYGFQPTNGVGYQ	8.00	WB	0.5299	5.50	WB	0.6057
**175**	**QSYGFQPTYGVGYQP**				**6.50**	**WB**	**0.7291**
**176**	**SYGFQPTYGVGYQPY**				**8.00**	**WB**	**0.8603**
**181**	**PTYGVGYQPYRVVVL**				**9.50**	**WB**	**0.6453**
182	TNGVGYQPYRVVVL	9.50	WB	0.5763	7.00	WB	0.8573
183	YGVGYQPYRVVVLSF				6.00	WB	1,0888
184	GVGYQPYRVVVLSFE				6.50	WB	1.3049
185	VGYQPYRVVVLSFEL				8.50	WB	1,3858
191	RVVVLSFELLHAPAT	9.50	WB	0.7485	6.00	WB	0.7485
192	VVVLSFELLHAPATV	4.50	WB	0.8083	3.00	WB	0.8083
193	VVLSFELLHAPATVC	3.00	WB	0.8618	2.50	WB	0.8618
194	VLSFELLHAPATVCG	1.80	SB	0.4784	1.70	SB	0.4784
195	LSFELLHAPATVCGP	1.70	SB	0.5062	1.60	SB	0.5062
196	SFELLHAPATVCGPK	2.50	WB	0.2085	1.90	SB	0.2085
197	FELLHAPATVCGPK	3.00	WB	0.0687	3.50	WB	0.1188
198	ELLHAPATVCGPKKS	8.50	WB	-0.1259			

Supplementary Table 4. In silico screening of MHC class I restricted T-cell based epitopes of the RBD protein from SARS-CoV-2. Using netMHCpan 4.1 MHC-I (H-2-D/K) restricted epitopes of 9 residues in length in the two more common mouse haplotypes (d and b) were selected and scored by Vaxijen 2.0. WB= Weak binding. SB= Strong Binding. New epitopes generated are colored in grey.

MHCI allelles

H-2-Dd

		ancestral RBD antigen			gamma RBD antigen		
**Position**	**Peptide**	**Score**	**Binding**	**VaxiJen**	**Score**	**Binding**	**VaxiJen**
6	ESIVRFPNI	0.695	WB	-0.1429 (NA)	0.695	SB	-0.1429 (NA)
9	VRFPNITNL	0.403	SB	1.114 (A)	0.403	WB	1.1141 (A)
24	FNATRFASV	0.975	WB	0.5609 (A)	0.975	WB	0.5609(A)
48	SVLYNSASF	1.63	WB	0.1857 (NA)	1.631	WB	0.1857 (NA)
51	YNSASFSTF	0.378	SB	0.3005 (NA)	0.378	SB	0.3005 (NA)
64	VSPTKLNDL	0.016	SB	1.4610 (A)	0.016	SB	1.4610 (A)
**92**	**IAPGQTGKI/ IAPGQTGTI**	0.197	SB	1.6527 (A)	0.098	SB	1.4132 (A)
**99**	**KIADYNYKL**	1.25	WB	1.6639 (A)			
103	YNYKLPDDF	1.45	WB	0.4828 (A)	1.453	WB	0.4828 (A)
126	KVGGNYNYL	1.77	WB	0.5994 (A)	1.772	WB	0.5994 (A)
129	GNYNYLYRL	0.895	WB	0.1170 (NA)	0.895	WB	0.1170 (NA)
**166**	**KGFNCYFPL**				0.944	WB	**0.5711 (A)**
**175**	**QSYGFQPTY**				1.124	WB	**1.1150 (A)**
177	YGFQPTNGV/YGFQPTYGV	1.79	WB	1.0509 (A)	1.449	WB	1.0317 (A)
185	VGYQPYRVV	0.279	SB	1.4383 (A)	0.279	SB	1.4383 (A)
187	YQPYRVVVL	0.056	SB	0.5964 (A)	0.056	SB	0.5964 (A)
**192**	**VVVLSFEL**	1.36	WB	1.2356 (A)	1.364	WB	1.0909 (A)
207	CGPKKSTNL	0.009	SB	0.1363 (NA)	0.009	SB	0.1363 (NA)

H-2-Kd

		ancestral RBD antigen			gamma RBD antigen		
**Position**	**Peptide**	**Score**	**Binding**	**VaxiJen**	**Score**	**Binding**	**VaxiJen**
9	VRFPNITNL	0.341	SB	1.1141 (A)	0.341	SB	1.1141 (A)
32	VYAWNRKRI	0.151	SB	0.5003 (A)	0.151	SB	0.5003 (A)
48	SVLYNSASF	1.966	WB	0.1857 (NA)	1.966	WB	0.1857 (NA)
50	LYNSASFST	1.936	WB	0.2718 (NA)	1.936	WB	0.2718 (NA)
61	CYGVSPTKL	0.108	SB	1.4263 (A)	0.108	SB	1.4263 (A)
81	SFVIRGDEV	0.808	WB	0.7549 (A)	0.808	WB	0.7549 (A)
126	KVGGNYNYL	1.677	WB	0.5994 (A)	1.677	WB	0.5994 (A)
130	NYNYLYRLF	1.424	WB	`-0.1480 (NA)	1.424	WB	`-0.1480 (NA)
154	IYQAGSTPC	1.077	WB	`-0.0319 (NA)	1.077	WB	`-0.0319 (NA)
170	CYFPLQSYG	1.475	WB	0.5780 (A)	1.475	WB	0.5780 (A)
186	GYQPYRVVV	0.066	SB	0.7634 (A)	0.066	SB	0.7634 (A)
**187**	**YQPYRVVV**	0.759	WB	0.2163 (A)	0.759	WB	0.5964 (A)

H-2-Ld

		ancestral RBD antigen			gamma RBD antigen		
**Position**	**Peptide**	**Score**	**Binding**	**VaxiJen**	**Score**	**Binding**	**VaxiJen**
3	QPTESIVRF	0.099	SB	`-0.5551 (NA)	0.099	SB	`-0.5551 (NA)
9	VRFPNITNL	1.69	SB	1.1141 (A)	1697	SB	1.1141 (A)
48	SVLYNSASF	1.57	WB	0.1857 (NA)	1.573	WB	0.1857 (NA)
51	YNSASFSTF	1.38	WB	0.3005 (NA)	1.382	WB	0.3005 (NA)
103	YNYKLPDDF	1.11	SB	0.4828 (A)	1118	SB	0.4828 (A)
**160**	**TPCNGVEGF/TPCNGVKGF**	0.64	WB	0.6322 (A)	1130	WB	-0.3226(NA)
191	RVVVLSFEL	0.997	WB	1.1918 (A)	0.997	WB	1.1918(A)
208	GPKKSTNLV	1.44	WB	0.6828 (A)	1.435	WB	0.6828 (A)

H-2-Db

		ancestral RBD antigen			gamma RBD antigen		
**Position**	**Peptide**	**Score**	**Binding**	**VaxiJen**	**Score**	**Binding**	**VaxiJen**
9	VRFPNITNL	0.368	SB	1.1141	0.368	SB	1,1141
48	SVLYNSASF	0.140	SB	0.1857	0.140	SB	0.1857
64	VSPTKLNDL	0.493	SB	1.4610	0.493	SB	1.4610
**92**	**IAPGQTGTI**				**0.613**	**WB**	**1,4132**
103	YNYKLPDDF	1.983	WB	0.4828	1.983	WB	0.4828
115	VIAWNSNNL	1.078	WB	0.7216	1.078	WB	0.7216
126	KVGGNYNYL	0.128	SB	0.5994	0.128	SB	0.5994
**166**	**KGFNCYFPL**				**1.296**	**WB**	**0.5711**
**177**	**YGFQPTYGV**				**1.927**	**WB**	**1.0317**
179	FQPTNGVGY	0.812	WB	0.3114			
187	YQPYRVVVL	0.258	SB	0.5964	0.258	SB	0.5964
191	RVVVLSFEL	0.910	WB	1,1918	0.910	WB	1,1918
192	VVVLSFELL	0.949	WB	1,0909	0.949	WB	1.0909
207	CGPKKSTNL	1.001	WB	0.1363	1.001	WB	0.1363

H-2-Kb

		ancestral RBD antigen		gamma RBD antigen			
**Position**	**Peptide**	**Score**	**Binding**	**VaxiJen**	**Score**	**Binding**	**VaxiJen**
6	ESIVRFPNI	0,614	<= WB	-0.1429	0.614	WB	-0.1429
9	VRFPNITNL	0,136	<= SB	1.1141	0.136	SB	1.1141
24	FNATRFASV	0	<= SB	0.5609	0.287	SB	0.5609
48	SVLYNSASF	1	<= WB	0.1857	0.963	WB	0.1857
51	YNSASFSTF	1,351	<= WB	0.3005	1351	WB	0.3005
64	VSPTKLNDL	0,384	<= SB	1.4610	0.384	SB	1,461
99	KIADYNYKL/TIADYNYKL	1	<= WB	1,663	0.630	WB	1,6035
115	VIAWNSNNL	1.038	<= WB	0.7216	1.038	WB	0.7216
126	KVGGNYNYL	1.728	<= WB	0.5994	1.728	WB	0.5994
129	GNYNYLYRL	0.036	<= SB	0.1170	0.036	SB	0.1170
**166**	**KGFNCYFPL**				**1046**	**WB**	**0.5711**
**175**	**QSYGFQPTY**				**0.847**	**WB**	**1.1150**
**177**	**YGFQPTYGV**				**1852**	**WB**	**1.0317**
185	VGYQPYRVV	0.132	<= SB	1,4383	0.132	SB	1,4383
187	YQPYRVVVL	1085	<= WB	0.5964	1085	WB	0.5964
192	VVVLSFELL	0.263	<= SB	1,0909	0.263	SB	1.0909
207	CGPKKSTNL	0.953	<= WB	0.1363	0.953	WB	0.1363

Supplementary Methods.

Prediction of restricted B and T-cell epitopes in the antigen sequences

Linear B-cell epitopes of the SARS-CoV-2 S protein were predicted by with a threshold of 0.5, and only the epitopes with a length between 5-25 residues were considered for subsequent antigenicity analysis. Antigenicity was evaluated via the VaxiJen v2.0 server online tool (VaxiJen v2.0., The Jenner Institute, Oxford, UK). Peptides with a score ≥ 0.4 were considered antigenic. Discontinuous B-cell epitopes were predicted via the DiscoTope 1.1 server tool in IEDB with a default threshold of −7.7 (corresponding specificity > 0.75 and

sensitivity < 0.47), based on the 3-dimensional (3D) structures of the SARS-CoV-2 RBD proteins obtained with Swiss model.

HTL- cell epitopes, were predicted based on the Net MHC pan 3.2 algorithm in IEDB. The 2 haplotypes chosen, H-2-IA$^{d/b}$ and H-2-IE$^{d/b}$, are common in laboratory mice strains. The algorithm was asked to predict peptides between 8 and 11 amino acids in length, for each haplotype. CD8^{+} T-cell epitopes were predicted based on the Net MHC pan 4.1 algorithm in IEDB, the peptide length selected was of 15 residues. As mentioned before, the haplotypes for the MHC class I molecules, were chosen according to the most prevalent in common laboratory mice strains (H-2-D$^{d/b}$, H-2-K$^{d/b}$). The prediction values are given in IC50 values (in nanoMolars) and as %Rank. The percentile rank for a peptide is generated by comparing its score against the scores of 200,000 random natural peptides of the same length of the query peptide. Strong and weak binding peptides are identified based on %Rank (Threshold strong binder: 2, Threshold weak binder: 10).
