# Supplementary material for: A Gamma-adapted subunit vaccine induces broadly neutralizing antibodies against SARS-CoV-2 variants and protects mice from infection
Source: Nat Commun. 2024 Feb 2;15:997. doi: 10.1038/s41467-024-45180-8 (PMC10837449; doi:10.1038/s41467-024-45180-8)
Supplement: Supplementary file 3 — Reporting Summary [file 41467_2024_45180_MOESM3_ESM.pdf]

nature portfolio

Corresponding author(s): Coria Lorena
Cassataro Juliana

Last updated by author(s): Dec 28, 2023

Reporting Summary

Nature Portfolio wishes to improve the reproducibility of the work that we publish. This form provides structure for consistency and transparency in reporting. For further information on Nature Portfolio policies, see our Editorial Policies and the Editorial Policy Checklist.

Statistics

For all statistical analyses, confirm that the following items are present in the figure legend, table legend, main text, or Methods section.

n/a	Confirmed	
☐	☒	The exact sample size (*n*) for each experimental group/condition, given as a discrete number and unit of measurement
☐	☒	A statement on whether measurements were taken from distinct samples or whether the same sample was measured repeatedly
☐	☒	The statistical test(s) used AND whether they are one- or two-sided<br>*Only common tests should be described solely by name; describe more complex techniques in the Methods section.*
☒	☐	A description of all covariates tested
☐	☒	A description of any assumptions or corrections, such as tests of normality and adjustment for multiple comparisons
☐	☒	A full description of the statistical parameters including central tendency (e.g. means) or other basic estimates (e.g. regression coefficient) AND variation (e.g. standard deviation) or associated estimates of uncertainty (e.g. confidence intervals)
☐	☒	For null hypothesis testing, the test statistic (e.g. *F*, *t*, *r*) with confidence intervals, effect sizes, degrees of freedom and *P* value noted<br>*Give P values as exact values whenever suitable.*
☒	☐	For Bayesian analysis, information on the choice of priors and Markov chain Monte Carlo settings
☒	☐	For hierarchical and complex designs, identification of the appropriate level for tests and full reporting of outcomes
☒	☐	Estimates of effect sizes (e.g. Cohen's *d*, Pearson's *r*), indicating how they were calculated

Our web collection on statistics for biologists contains articles on many of the points above.

Software and code

Policy information about availability of computer code

Data collection

Antibody titers were deterimined by ELISA using a 800TS microplate reader (BioTek instrument Ink.) with Gen5 software v3.10.06 (Biotek Instrument Ink.).
In neutralization assays, the cytopathic effect (CPE) of the virus o was assessed by surface scanning in the FilterMAx F5 Microplate reader Molecular Devices, San Jose, CA, USA) with the Softmax PRO software. Version 7.1.
Flow cytometry analysis was performed in the BD Fortessa LSR-X20 Cytometer with BD FACSDiva Software (Version 8.0.2).

Data analysis

Flow cytometry files analysis was performed with FlowJo X Version 10..8.1.
Cytokines in culture supernatans were measured by flow cytometry with the multiplex bead assay and data analysis was done with the LEGENDplex™ Data Analysis Software Suite. Version Version 2023-02-15.
Graphs and statistical analyisis was done with GraphPad Prism version 8.
Generation of antigenic maps were done using the R package Racmacs (https://acorg.github.io/Racmacs/index.html).
Prediction of linear B-cell epitopes was performed with BepiPred 2.0 in IEDB (BepiPred 2.0., Immune Epitope Database and Analysis Resource, National Institute of Allergy and Infectious Diseases, Bethesda, MD, USA).
Discontinuous B-cell epitopes were predicted via the DiscoTope 1.1 server tool in IEDB
T cell epitopes, were predicted based on the Net MHC pan 3.2 and Net MHC pan 4.1 algorithm in IEDB
The R package Racmacs (https://acorg.github.io/Racmacs/index.html) was used to create antigen cartography maps from serum neutralization titers against the SARS-CoV-2 live viruses .

For manuscripts utilizing custom algorithms or software that are central to the research but not yet described in published literature, software must be made available to editors and reviewers. We strongly encourage code deposition in a community repository (e.g. GitHub). See the Nature Portfolio guidelines for submitting code & software for further information.

Data

Policy information about availability of data

All manuscripts must include a data availability statement. This statement should provide the following information, where applicable:

- Accession codes, unique identifiers, or web links for publicly available datasets
- A description of any restrictions on data availability
- For clinical datasets or third party data, please ensure that the statement adheres to our policy

We included a data availability statement: "All data supporting the findings of this study are available within the paper. Source Data are provided with this paper. Any additional information related to the study also is available from the corresponding author upon reasonable request."
We provided the Supplementary information and Source Data files that contains all the data to interpret and verify the research presented in our article.
If extra data is needed by the readers, it can be requested to the corresponding author.

Research involving human participants, their data, or biological material

Policy information about studies with human participants or human data. See also policy information about sex, gender (identity/presentation), and sexual orientation and race, ethnicity and racism.

Reporting on sex and gender	*Use the terms sex (biological attribute) and gender (shaped by social and cultural circumstances) carefully in order to avoid confusing both terms. Indicate if findings apply to only one sex or gender; describe whether sex and gender were considered in study design; whether sex and/or gender was determined based on self-reporting or assigned and methods used. Provide in the source data disaggregated sex and gender data, where this information has been collected, and if consent has been obtained for sharing of individual-level data; provide overall numbers in this Reporting Summary. Please state if this information has not been collected. Report sex- and gender-based analyses where performed, justify reasons for lack of sex- and gender-based analysis.*
Reporting on race, ethnicity, or other socially relevant groupings	*Please specify the socially constructed or socially relevant categorization variable(s) used in your manuscript and explain why they were used. Please note that such variables should not be used as proxies for other socially constructed/relevant variables (for example, race or ethnicity should not be used as a proxy for socioeconomic status). Provide clear definitions of the relevant terms used, how they were provided (by the participants/respondents, the researchers, or third parties), and the method(s) used to classify people into the different categories (e.g. self-report, census or administrative data, social media data, etc.) Please provide details about how you controlled for confounding variables in your analyses.*
Population characteristics	*Describe the covariate-relevant population characteristics of the human research participants (e.g. age, genotypic information, past and current diagnosis and treatment categories). If you filled out the behavioural & social sciences study design questions and have nothing to add here, write "See above."*
Recruitment	*Describe how participants were recruited. Outline any potential self-selection bias or other biases that may be present and how these are likely to impact results.*
Ethics oversight	*Identify the organization(s) that approved the study protocol.*

Note that full information on the approval of the study protocol must also be provided in the manuscript.

Field-specific reporting

Please select the one below that is the best fit for your research. If you are not sure, read the appropriate sections before making your selection.

☒ Life sciences ☐ Behavioural & social sciences ☐ Ecological, evolutionary & environmental sciences

For a reference copy of the document with all sections, see nature.com/documents/nr-reporting-summary-flat.pdf

Life sciences study design

All studies must disclose on these points even when the disclosure is negative.

Sample size	Sample size was determined by previous experience measuring antibody and cellular immune respones in vaccinated and unvaccinated mice PMID: 35296091. A minimum of five animals per group was used in vaccinated groups that represents a sufficient sample size to detect statistical differences between experimental groups. No sample size calculations were performed prior to data collection.
Data exclusions	Exclusion of data was done when samples were no optimal for analysis. In flow cytometry data from figure 4a, one sample of the gamma RBD plus alum group presented has a major percentage of death cells. We excluded the data from that animal. In Fig 4b, ancestral vaccine group incubated with medium sample from one mice was also excluded in IL-17A and IL-22 graphs.
Replication	Most of the experiments has two or three biological independent repeats and are indicated in the figure legends. Similar findings were obtained from all repeats. Only one experiment (Figure 3c-d) was performed once because involved data collection until day 253 after immunization.

Randomization

Mice were randomly assigned to each cage in age equal distribution by personnel from the animal facility at IIB-UNSAM. Then, each group (placebo or vaccinated animals) was randomly assigned to each cage. In challenge experiments equal sex distribution was performed per group. All data presented in the article came from animal samples. There were not any other experimental procedure that requires randomization.

Blinding

Blinding was not possible during animal manipulations and immunizations because we received labelled vaccine vials. Neutralizing assays were performed by a technician in a blinded manner. Also, blinding was performed for the histopathologists that analyzed lung tissues. Experiments to evaluate cellular immune responses were performed by different personnel from the lab in a blinded manner.

Reporting for specific materials, systems and methods

We require information from authors about some types of materials, experimental systems and methods used in many studies. Here, indicate whether each material, system or method listed is relevant to your study. If you are not sure if a list item applies to your research, read the appropriate section before selecting a response.

Materials & experimental systems

n/a	Involved in the study	
☐	☒	Antibodies
☐	☒	Eukaryotic cell lines
☒	☐	Palaeontology and archaeology
☐	☒	Animals and other organisms
☒	☐	Clinical data
☒	☐	Dual use research of concern
☒	☐	Plants

Methods

n/a	Involved in the study	
☒	☐	ChIP-seq
☐	☒	Flow cytometry
☒	☐	MRI-based neuroimaging

Antibodies

Antibodies used

List of all antibodies used in the study:
ELISA to determinant anti-RBD total IgG antibodies
1- HRP conjugated anti-mouse IgG . Dilution 1/2000. (SIGMA, Cat #A4416-1ML, clone Polyclonal) PMID: 34745450.
Antibodies used in the ACE2 binding ELISA.
2- anti-hACE2 (R&D Systems, Catalog#: AF933. Lot: HOK0620051) diluted to a concentration of 0.5µg/ml.
3- HRP (Agilent DAKO, Catalog# P0449, Lot: 41308941) at a dilution: 1/1000
Flow cytometry antibodies:
4- Alexa Fluor 488 anti-B220 (Biolegend, Cat #103225, clone RA3-6B2, 1/400). PMID: 32699279.
5- APC/Cy7 anti-CD19 (Biolegend, Cat #115530, clone 6D5, 1/200). PMID: 32601467.
6- Brilliant Violet 785 anti-CD138 (Biolegend, Cat #142534, clone 281-2, 1/200). PMID: 33521747.
7- Brilliant Violet 605 anti-IgD (Biolegend, Cat #405727, clone 11-26c2a, 1/150). PMID: 34852217.
8- PECy7 anti-IgG (Biolegend, Cat #405315, clone Poly4053, 1/200). PMID: 34195186.
9- Alexa Fluor 594 anti-B220 (Biolegend, Cat #103254, clone RA3-6B2, 1/100). PMID: 33298977.
10- Alexa Fluor 488 anti-GL7 (Biolegend, Cat #144612, clone GL7, 1/200). PMID: 36115859.
11- PE anti-CD95 (Biolegend, Cat #152608, clone SA36758, 1/800). PMID: 33767434.
12- Brilliant Violet 711 anti-CD4 (Biolegend, Cat #100447, clone GK1.5, 1/400). PMID: 33296701.
13- Alexa Fluor 700 anti-CD8 (Biolegend, Cat #100730, clone 53-6.7, 1/200). PMID: 31926851.
14- PECy7 anti-CXCR5 (Biolegend, Cat #145516, clone L138D7, 1/200). PMID: 34326343.
15- Brilliant Violet 421 anti-PD-1 (Biolegend, Cat #135221, clone 29F.1A12, 1/100). PMID: 33789089.
16- Multiplex bead assay LEGENDplex™ MU Th Cytokine Panel (12-plex) (Biolegend, Cat # 741043). PMID: 34359702.
17- Alexa Fluor 488 anti-mouse-CD8a (Biolegend, Cat #100723, clone 53-6.7, 1/100). PMID: 33212014.
18- Alexa Fluor 647 anti-mouse-CD4 (Biolegend, Cat #100424, clone 6K1.5, 1/200). PMID: 31053504.
19- PECy7 anti-TNFα (Biolegend, Cat #506324, clone MP6-XT22, 1/50). PMID: 34433030.
20- PE anti-IFN-γ (Biolegend, Cat #505808, clone XMG1.2, 1/50). PMID: 24631089.
21- Brilliant Violet 421 anti-IL-2 (Biolegend, Cat #503826, clone JES6-5H4, 1/50). PMID: 35739199.
22- Ultra-LEAF™ Purified anti-mouse CD49d Antibody (Biolegend, Cat #103710, clone 9C10, 1/1000). PMID: 33257686.
23- Ultra-LEAF™ Purified anti-mouse CD28 Antibody (Biolegend, Cat #103710, clone 9C10, 1/1000). PMID: 32877667.
Antibodies used in the Western blot of Figure 1a:
24- In house anti-RBD rabbit polyclonal serum (1/1000 dilution).
25-anti-rabbit IgG horseradish-peroxidase conjugated (1/2000 dilution). Agilent DAKO.Catalog# P0448, Lot: 20061231.

Validation

Commercial antibodies were validated by the manufacturers and validation statements are available on the manufacturer's website.
Statement from BioLegend: Specificity testing of 1-3 target cell types with either single- or multicolor analysis (including positive and negative cell types). Once specificity is confirmed,each new lot must perform with similar intensity to the in-date reference lot. Brightness (MFI) is evaluated from both positive and negative populations. Each lot product is validated by QC testing with a series of titration dilutions.
Sigma-aldrich. HRP conjugated anti-mouse IgG . Cat #A4416-1ML. Due to product improvement and changes in the assay procedure, a lot specific titer by direct ELISA for this product is provided. Due to differences in assay systems, this titer may not reflect the user's actual working dilution.
Additional literature related with validation of antibodies can be found in the manufacturer´s website

Eukaryotic cell lines

Policy information about cell lines and Sex and Gender in Research

Cell line source(s)	VERO E6 cells, source ATCC. A cell bank was generated at UNSAM
Authentication	Cell line in continous use and used for SARS-CoV-2 neutralization assays performed at UNSAM. No extra authentication was performed.
Mycoplasma contamination	The Vero E6 cell line tested negative for mycoplasma contamination. Mycoplasma testing is routinely performed.
Commonly misidentified lines (See ICLAC register)	No commonly misidentified lines were used in the study.

Animals and other research organisms

Policy information about studies involving animals; ARRIVE guidelines recommended for reporting animal research, and Sex and Gender in Research

Laboratory animals	Eight-week-old female BALB/c (BALB/cJ) or C57BL/6 (C57BL/6J) were used for immunogenicity studies. Mice were obtained from The Jackson Laboratory and were bred in the animal facility of IIB-UNSAM.<br>In the omicron BA.5 challenge experiment, transgenic B6.Cg-Tg(K18-ACE2)2Prlmn/J (K18-hACE2, JAX stock #034860) were purchased from Jackson Laboratories (Bar Harbor, Maine, USA), transported to Argentina and housed and bred at the Animal Facility of the Center for Comparative Medicine (ICIVET-Litoral, UNL-CONICET). Four month-old K18-hACE2 mice (N=12/per group, 6 males and 6 females per group) were used.<br>In the wuhan SARS-CoV-2 challgenge experiment, four-week-old transgenic B6.Cg-Tg(K18-ACE2)2Prlmn/J mice from Jackson Laboratory were used. Animals were housed at the Department of Entomology, College of Agriculture and Life Sciences, Fralin Life Science Institute, Virginia Polytechnic Institute. n=8 mice (placebo group) n= 9 mice in vaccinated group, equal amount of males and females per group.<br>Animals in all animal facilities have a 12h dark/12h light cycle. Ambient temperature 21-23°C and 55% (±5) humidity. Animals have accest to food and water ad libitum.
Wild animals	The study did not involve wild animals.
Reporting on sex	In challenge experiments mice from both sexes (female and male) were distributed equally in each experimental group. No sex-based analysis was performed.<br>In immunogenicity studies only female mice were used. Sex in these studies was considered in terms of animal behaviour as male are more agressive and need to be in separated cages. We do not have the available space in the animal facility to use animals of both sexes in all experiments.
Field-collected samples	The study did not involve samples collected from the field.
Ethics oversight	All experimental protocols with animals were conducted in strict accordance with international ethical standards for animal experimentation (Helsinki Declaration and its amendments, Amsterdam Protocol of welfare and animal protection and National Institutes of Health, NIH USA, guidelines.). The protocols performed were also approved by the Institutional Committee for the use and care of experimental animals (CICUAE) from National University of San Martin (UNSAM) (Protocol number 01/2020). All procedures involving animals at Virginia Tech were approved on 04/09/2021 by the Virginia Tech's Institutional Animal Care and Use Committee (IACUC) and all animal experiments were performed in compliance with the guidelines of Virginia Tech's IACUC. Animal procedures at the Universidad del Litoral were approved by the Ethics Committee of the Facultad de Ciencias Veterinarias, Universidad Nacional del Litoral, Santa Fe, Argentina (Protocol number 808/23).

Note that full information on the approval of the study protocol must also be provided in the manuscript.

Flow Cytometry

Plots

Confirm that:

☒ The axis labels state the marker and fluorochrome used (e.g. CD4-FITC).

☒ The axis scales are clearly visible. Include numbers along axes only for bottom left plot of group (a 'group' is an analysis of identical markers).

☒ All plots are contour plots with outliers or pseudocolor plots.

☒ A numerical value for number of cells or percentage (with statistics) is provided.

Methodology

Sample preparation	All samples were from mouse organs. Spleen or lymph nodes cell suspension were obtained by filtering the organs with a 100um mesh with RPMI medium containing 2% serum. Then cells were counted and labeled with Zombie Acqua to determine live cells. Then cells were washed and Fc receptors were blocked with TruXstain (Biolegend) for 15 minutes. Afterwards, cells were stained with appropriate mixed fluorescent antibodies and/or proteins for 30 min in ice. Then, cells

were washed twice and fixed with 1% paraformaldehide for 20 min.
For the determination of cytokines in cell culture supernatants the instructions of manufacturer (Legendplex, Biolegend) was followed.

Instrument

BD Fortessa LSR X-20, BD Biosciences.

Software

Data collection was done with the Diva Software (BD Biosciences). Analysis was performed with FlowJo X software Version 10.8.1 (FlowJo, LLC).

Cell population abundance

No sorting populations were used.

Gating strategy

All gating strategies are shown in the supplementary information file.

Gating strategy for analysis of B cells and plasmablasts in spleen:
1.FSC-W vs. FSC-A for selection of single cells
2.FSC-A vs SSC-A for selection of lymphocytes
3.FSC-A vs Zombie acqua for selection of live cells
4.B220 vs CD19 for selection of B cells
5. CD19 vs IgD for selection of IgD negatives B cells:
6a.RBD vs CD19 for selection of B cells specifics for RBD
6b. CD138 vs B220 for seleciton of plasmablasts and then RBD vs B220 for selection of specific plasmablasts
6c. IgG vs B220 for seleciton of B cells that express IgG and then BD vs B220 for selection of specific B cells that express IgG.
Fluoresence minus one (FMO) were used to determine the RBD positive populations.

Gating strategy for analysis of germinal centre B cells:
1.FSC-W vs. FSC-A for selection of single cells
2.FSC-A vs SSC-A for selection of lymphocytes
3.FSC-A vs Zombie acqua for selection of live cells
4.B220 vs CD19 for selection of B cells
5. GL7 vs CD95 for selection of germinal centre B cells.

Gating strategy for analysis of germinal centre B cells:
1.FSC-W vs. FSC-A for selection of single cells
2.FSC-A vs SSC-A for selection of lymphocytes
3.FSC-A vs Zombie acqua for selection of live cells
4. CD4 vs SSC-A for selection of CD4 T cell population
5. PD-1 vs CXCR5 for selection of T follicular helper cells.

Gating strategy for analysis of long live plasma cells (LLPCs):
1.FSC-W vs. FSC-A for selection of single cells
2.FSC-A vs SSC-A for selection of lymphocytes
3.FSC-A vs Zombie acqua for selection of live cells
4. CD138 vs B220 for selection of LLPCs
5a. RBD vs B220 for selection of specific LLPCs
5b. IgG vs B220 for selecion of IgG expressing LLPCs and then RBD vs B220 for selection of specific cells.
Fluoresence minus one (FMO) were used to determine the RBD positive populations.

Gating strategy for analisis of intracellular flow cytometry:
1.FSC-W vs. FSC-A for selection of single cells
2.FSC-A vs SSC-A for selection of lymphocytes
3.FSC-A vs Zombie acqua for selection of live cells
4. CD4 vs CD8 for selection of CD4 or CD8 T cell populations
5. CD4 vs IFN-g, CD4 vs TNF-a or CD4 vs IL-2 for selection of CD4 T cells expressing cytokines.
5. CD8 vs IFN-g, CD8 vs TNF-a or CD8 vs IL-2 for selection of CD8 T cells expressing cytokines.
Fluoresence minus one (FMO) were used to determine cytokines positive populations.

☒ Tick this box to confirm that a figure exemplifying the gating strategy is provided in the Supplementary Information.
